# Construction and Immunogenicity Evaluation of Recombinant Influenza A Viruses Containing Chimeric Hemagglutinin Genes Derived from Genetically Divergent Influenza A H1N1 Subtype Viruses

**DOI:** 10.1371/journal.pone.0127649

**Published:** 2015-06-10

**Authors:** Kara McCormick, Zhiyong Jiang, Longchao Zhu, Steven R. Lawson, Robert Langenhorst, Russell Ransburgh, Colin Brunick, Miranda C. Tracy, Heather R. Hurtig, Leah M. Mabee, Mark Mingo, Yanhua Li, Richard J. Webby, Victor C. Huber, Ying Fang

**Affiliations:** 1 Division of Basic Biomedical Sciences, Sanford School of Medicine, The University of South Dakota, Vermillion, SD, 57069, United States of America; 2 Department of Veterinary and Biomedical Sciences, South Dakota State University, Brookings, SD, 57007, United States of America; 3 Department of Infectious Diseases, St. Jude Children’s Research Hospital, Memphis, TN, 38105, United States of America; Virginia Polytechnic Institute and State University, UNITED STATES

## Abstract

**Background and Objectives:**

Influenza A viruses cause highly contagious diseases in a variety of hosts, including humans and pigs. To develop a vaccine that can be broadly effective against genetically divergent strains of the virus, in this study we employed molecular breeding (DNA shuffling) technology to create a panel of chimeric HA genes.

**Methods and Results:**

Each chimeric HA gene contained genetic elements from parental swine influenza A viruses that had a history of zoonotic transmission, and also from a 2009 pandemic virus. Each parental virus represents a major phylogenetic clade of influenza A H1N1 viruses. Nine shuffled HA constructs were initially screened for immunogenicity in mice by DNA immunization, and one chimeric HA (HA-129) was expressed on both a A/Puerto Rico/8/34 backbone with mutations associated with a live, attenuated phenotype (PR8_LAIV-_129) and a A/swine/Texas/4199-2/98 backbone (TX98-129). When delivered to mice, the PR8_LAIV-_129 induced antibodies against all four parental viruses, which was similar to the breadth of immunity observed when HA-129 was delivered as a DNA vaccine. This chimeric HA was then tested as a candidate vaccine in a nursery pig model, using inactivated TX98-129 virus as the backbone. The results demonstrate that pigs immunized with HA-129 developed antibodies against all four parental viruses, as well as additional primary swine H1N1 influenza virus field isolates.

**Conclusion:**

This study established a platform for creating novel genes of influenza viruses using a molecular breeding approach, which will have important applications toward future development of broadly protective influenza virus vaccines.

## Introduction

Influenza A viruses infect a variety of avian and mammalian hosts, including humans and pigs, and thus pose a significant pandemic threat [1]. Vaccines against influenza viruses are available for both pigs and humans, with human vaccines receiving annual updates based on surveillance [2]. These vaccines are designed to limit transmission and infection with host species-restricted variants within a single influenza A virus subtype [3,4], and they demonstrate efficacy within their respective populations [5,6]. However, sporadic transmissions of influenza A viruses across species barriers have been noted historically [7], with some of these events being associated with human pandemics [8,9]. Since 2009, the emergence and pandemic classification of a triple reassortant influenza A virus (H1N1 subtype) containing swine, human and avian genetic components raised greater concerns over future pandemics of swine-origin viruses. Specifically, there is a possibility that novel viruses could evolve within swine populations to yield viruses with increased transmissibility and virulence within humans [10]. Since vaccination remains the primary means for controlling seasonal influenza viruses, combining our efforts to limit interspecies transmission events represents a potential path toward a pandemic vaccine. A vaccine that could limit the circulation of influenza viruses among pigs, as well as prevent interspecies transmission events from pigs to humans, would strengthen these efforts.

Seasonal influenza vaccines have historically demonstrated moderate effectiveness when the circulating strains closely match the vaccine strain [6], but the success of the vaccine can be compromised when there is not a close match [5,11]. Efforts to generate vaccines that match circulating strains can be time-consuming [12], and in pigs the reformulation of swine influenza vaccines can be limited by the surveillance data available [13]. Thus, a vaccine that can induce strong, broad, protective immunity toward multiple heterologous strains is urgently needed in both pigs and humans. A previous study by our group [14] reported that multiple, individual human influenza A virus hemagglutinins (HAs), from the H3N2 subtype, could be delivered simultaneously to induce immunity that covered approximately 20 years of HA evolution. This proof-of-concept approach showed that broad immunity can be achieved, within an influenza A virus subtype. However, when these distinct HAs were delivered by simultaneously inoculating with multiple whole virus preparations, antibody titers were not detected against all of the HAs included in the vaccine [14]. Thus, improvement on this approach is needed.

A molecular breeding (DNA shuffling) strategy represents a novel approach to produce broadly protective vaccines. DNA shuffling is a process of random recombination of parental genes into novel genes, with shuffled (recombined) chimeric genes being selected for desired properties [15–23]. The importance of this process is that molecular breeding by DNA shuffling of specific genes mimics the evolution pathway and accelerates the natural process of evolution for viruses, or viral proteins, *in vitro* [24]. In this study, we applied molecular breeding technology toward producing a vaccine against influenza A virus in pigs. Since the viral surface glycoprotein HA has been the major target of most licensed influenza vaccines, we specifically targeted the HA from the 2009 pandemic virus, as well as HAs from three additional swine influenza viruses that had a history of zoonotic transmission to humans [25,26]. These parental influenza A H1N1 strains represent four distinct phylogenetic clades, and HA genes of these four parental strains were used for DNA shuffling and screening to generate a panel of chimeric influenza HA antigens. One chimeric construct, HA-129, was further presented in the context of a traditional, whole virus vaccine backbone, and immune responses induced by this chimera were evaluated in both mice and pigs. Results from this study suggest that chimeric HA antigens generated by DNA shuffling would have potential applications as broadly protective influenza vaccines.

## Materials and Methods

### Parental HA genes and viral strains

The HA genes of the four parental H1N1 influenza A viruses A/Tennessee/1-560/09 (TN09; CY040457.1), A/New Jersey/8/1976 (NJ76; CY130118.1), A/Ohio/01/2007 (OH07; FJ986620.1), and A/Iowa/01/2006 (IA06; FJ986618.1) were amplified by RT-PCR from stock viruses using the Bm-HA-1F (TATTCGTCTCAGGGAGCAAAAGCAGGGG) and Bm-NS-890R (ATATCGTCTCGTATTAGTAGAAACAAGGGTGTTTT) primers, with PCR products cloned in pHW2000 plasmid using *Bsm*BI [27]. Additional H1N1 influenza A virus strains that were tested to demonstrate cross reactivity include A/North Carolina/18161/2002 (NC02; CY098516.1), A/swine/Iowa/1/1985 (IA85; CY022317.1), A/swine/Iowa/40766/1992 (IA92; KP788773), A/swine/Germany/2/1981 (GE81; Z30276.1), and A/New Caledonia/20/99 (NC99; CY125100.1).

### DNA shuffling of HA genes

The DNA shuffling of HA genes was performed as described by Soong et al [28], with minor modifications. Briefly, DNA products of HA genes from the four parental strains (TN09, NJ76, OH07, and IA06) were mixed equimolarly and digested with DNase I. The DNA fragments were assembled as described previously [24], and the reassembled fragments were amplified by PCR using the Bm-HA-1F and Bm-NS-890R primers. The PCR products were cloned into the pHW2000 plasmid to establish the chimeric HA library.

### Creation and characterization of HA-expressing virus reassortants

The 8-plasmid reverse genetics system, incorporating co-cultured 293T (American Type Culture Collection, Manassas, VA) and MDCK (ATCC) cells, was used to create reassortant viruses in this study. For viruses expressing the cloned parental HA genes from TN09, OH07, NJ76, or IA06, the viruses were created using reverse genetics, with each HA incorporated into a reassortant virus that derived the 7 other influenza virus genes from the A/Puerto Rico/8/34 (PR8) donor virus [29]. Viruses rescued from 293T:MDCK cell co-cultures that expressed the desired HA were propagated in 10-day-old embryonated chicken eggs for 72 h at 35°C and sequenced to verify appropriate HA genotype. Similarly, when expressing the chimeric HA construct, HA-129, within influenza viruses for vaccine creation, we used the live, attenuated influenza virus (LAIV) PR8 (PR8_LAIV_) backbone for generating a candidate vaccine in mouse (PR8_LAIV_-129), while the A/swine/Texas/4199-2/98 swine reverse genetics system was used for generating a candidate inactivated influenza virus (IIV) vaccine in pigs (TX98-129) [30].

The vaccine virus rescued on the PR8_LAIV_ backbone (PR8_LAIV_-129) was propagated in 10 day-old embryonated chicken eggs for 72 h at 33°C, as described previously [14,31], and the TX98-129 virus was propagated for 72 h at 35°C. The growth characteristics of these viruses were determined using MDCK cells, as previously described [32]. Briefly, MDCK cell monolayers (3x10^5^ cells per well) were inoculated with influenza viruses in the presence of TPCK-trypsin, and at indicated times, amounts of virus present were tested using standard methods for calculating the median tissue culture infectious dose (TCID_50_) of influenza viruses [33].

### Mice and immunization

Adult (6–8-week-old) female BALB/cJ mice were obtained from Harlan Laboratories (Indianapolis, IN) and housed in groups of four, with 24-hour access to food and water. All mouse experiments were performed following the guidelines established and approved by the Animal Care and Use committee at the University of South Dakota (Vermillion, SD). For DNA immunization, plasmid DNA was coupled to gold particles as described previously [34], and administered directly to the mouse abdomen, using a Helios gene gun (Bio-Rad Laboratories, Hercules, CA). Mice were boosted twice with a 3-week interval between inoculations. Three weeks after the third inoculation, sera were collected and analyzed by ELISA. For whole virus vaccination, mice that were lightly anesthetized with 2.5% isoflurane were inoculated with 1x10^5^ TCID_50_ PR8_LAIV_-129 in a 50μl volume, and boosted with 1x10^5^ TCID_50_ PR8_LAIV_-129 at 28 days post inoculation (dpi). Sera were collected at 21 days after the second inoculation with whole virus. To inactivate host innate immune inhibitors of influenza virus, sera were treated with receptor-destroying enzyme (RDE, Accurate Chemical, Westbury, NY) and heat-inactivated as described previously [35].

### Antibody detection by ELISA

Serum antibodies were detected using an ELISA, as described previously [14]. Briefly, 96-well flat bottom plates (NUNC, Thermo Fisher Scientific, Waltham, MA) were coated with concentrated, formalin-inactivated parental viruses (1 μg HA mL^−1^). RDE-treated sera were serially diluted in PBS containing 10% fetal bovine serum (FBS) (Atlanta Biologicals, Lawrenceville, GA) and 0.05% (v/v) Tween-20 (Sigma, St. Louis, MO) (FBS-PBST). Alkaline phosphatase-conjugated preparations of goat anti-mouse IgG (γ-specific) antibodies (Southern Biotechnology, Inc., Birmingham, AL), diluted in FBS-PBST, were added to the plate. Plates were washed, and 1 mg mL^−1^ *p*-nitrophenyl phosphate substrate (Sigma) in diethanolamine buffer was added. One hour after substrate addition, the OD was detected at 405 nm using a BioTek EL808 plate reader (BioTek Instruments, Inc., Winooski, VT). Reciprocal serum antibody titers for individual serum samples are reported at 50% maximal binding on the individual titration curves. Individual sera were considered positive only if their starting dilution OD_405_ values were greater than 3 times the OD_405_ of negative control sera. Samples that did not show a detectable titer at the starting serum dilution of 1:50 were assigned a titer of 50 for the purpose of graphing.

### Hemagglutination Inhibition and Microneutralization assays

Hemagglutination inhibition (HAI) assays were performed as described previously [35]. Briefly, RDE-treated sera were diluted serially, and four HA units of virus were added to each well. The virus:sera mixtures were incubated for one hour at 4°C, at which time a solution of 0.5% solution of chicken red blood cells (Lampire Biological Laboratories, Pipersville, PA) was added to each well. Titers are reported as the reciprocal of the final serum dilution that inhibited hemagglutination. Similarly, microneutralization (MN) assays were performed as previously described [32,35], using 100 TCID_50_ for each virus inoculated onto confluent MDCK monolayers. Infected MDCK cells were identified using monoclonal antibodies against the influenza A virus nucleoprotein, with a titer defined as the last dilution that inhibited detection of NP below 50% of the OD_490_ for positive control wells, as described previously [36,37]. For both HAI and MN assays, serum samples that did not show a detectable titer at the starting serum dilution of 1:10 were assigned a titer of 5 for the purpose of both graphing and statistical analyses.

### Nursery pig study

Nursery pigs (3 weeks old) that were free of swine influenza virus, porcine reproductive and respiratory syndrome virus, and *Mycoplasma hyopneumoniae* were obtained. They were randomly divided into three groups, and housed separately in animal isolation facilities at South Dakota State University (SDSU). All pig experiments were performed following the guidelines established and approved by the Animal Care and Use committee at South Dakota State University (Brookings, SD). In contrast to the murine model, live influenza virus was not given to pigs, due to biosafety considerations. Therefore, TX98-129 virus was formalin-inactivated as described previously [38], and pigs were immunized intramuscularly with 100 μg/ml of inactivated virus in a 2 mL volume. As a negative control, an unvaccinated group of pigs was inoculated with a similar volume of PBS. Fourteen days after primary inoculation, pigs were boosted with the same dose of antigen, and sera were collected and analyzed at 14 days post-secondary inoculation.

### Data analysis

DNASTAR and MEGA4 were used for sequence alignment and phylogenetic analyses. Table 1 lists all the influenza virus strains from which HA genes were used for constructing the phylogenetic tree. Analysis of HA chimeras created by DNA shuffling was performed by using the Salanto method (*https*:*//bitbucket*.*org/benderc/salanto/wiki/Home*). Two way analysis of variance and nonparametric Mann Whitney tests were used to analyze the data. Significant differences between groups were evaluated using Bonferroni post-tests. All statistical analyses were performed using either JMP 5.1 (SAS Institute, Cary, NC) or GraphPad Prism version 4.00 for Windows (GraphPad Software, San Diego California USA, *www.graphpad.com*).

**Table 1:** Virus Names, Subtypes and Accession Numbers Included in Phylogenetic Tree

Virus Name	Accession
A/Ohio/01/2007(H1N1)	FJ986620
A/swine/Minnesota/03025/2010(H1N1)	HM570051
A/swine/Illinois/03037/2010(H1N1)	HM754221
A/Swine/Ohio/891/01(H1N2)	AF455675
A/Tennessee/1-560/2009(H1N1)	CY040457
A/New Jersey/8/1976(H1N1)	CY130118
A/Texas/05/2009(H1N1)	FJ966959
A/California/04/2009(H1N1)	FJ966082
A/Iowa/01/2006(H1N1)	FJ986618
A/swine/Kentucky/02086/2008(H1N1)	HM461786
A/swine/Iowa/1973(H1N1)	EU139826
A/swine/Iowa/2/1987(H1N1)	CY028171
A/swine/Ontario/53518/03(H1N1)	DQ280219
A/swine/Minnesota/02053/2008(H1N1)	CY099119
A/swine/Iowa/1/1985(H1N1)	CY022317
A/Swine/North Carolina/98225/01(H1N2)	AF455676
A/Swine/Iowa/930/01(H1N2)	AF455679
A/swine/MN/48683/2002(H1N1)	HM125974
A/swine/North Carolina/18161/2002(H1N1)	CY098516
A/swine/Germany/2/1981(H1N1)	Z30276
A/swine/Tennessee/49/1977(H1N1)	CY022133
A/swine/Tennessee/8/1978(H1N1)	CY027523
A/swine/Netherlands/12/85(H1N1)	AF091317
A/South Carolina/1/18(H1N1)	AF117241
A/swine/Iowa/15/1930(H1N1)	EU139823
A/swine/Colorado/1/1977(H3N2)	CY009300
A/swine/Iowa/40766/1992(H1N1)	KP788773

## Results

### Construction and screening of chimeric HA genes

**Fig 1:**
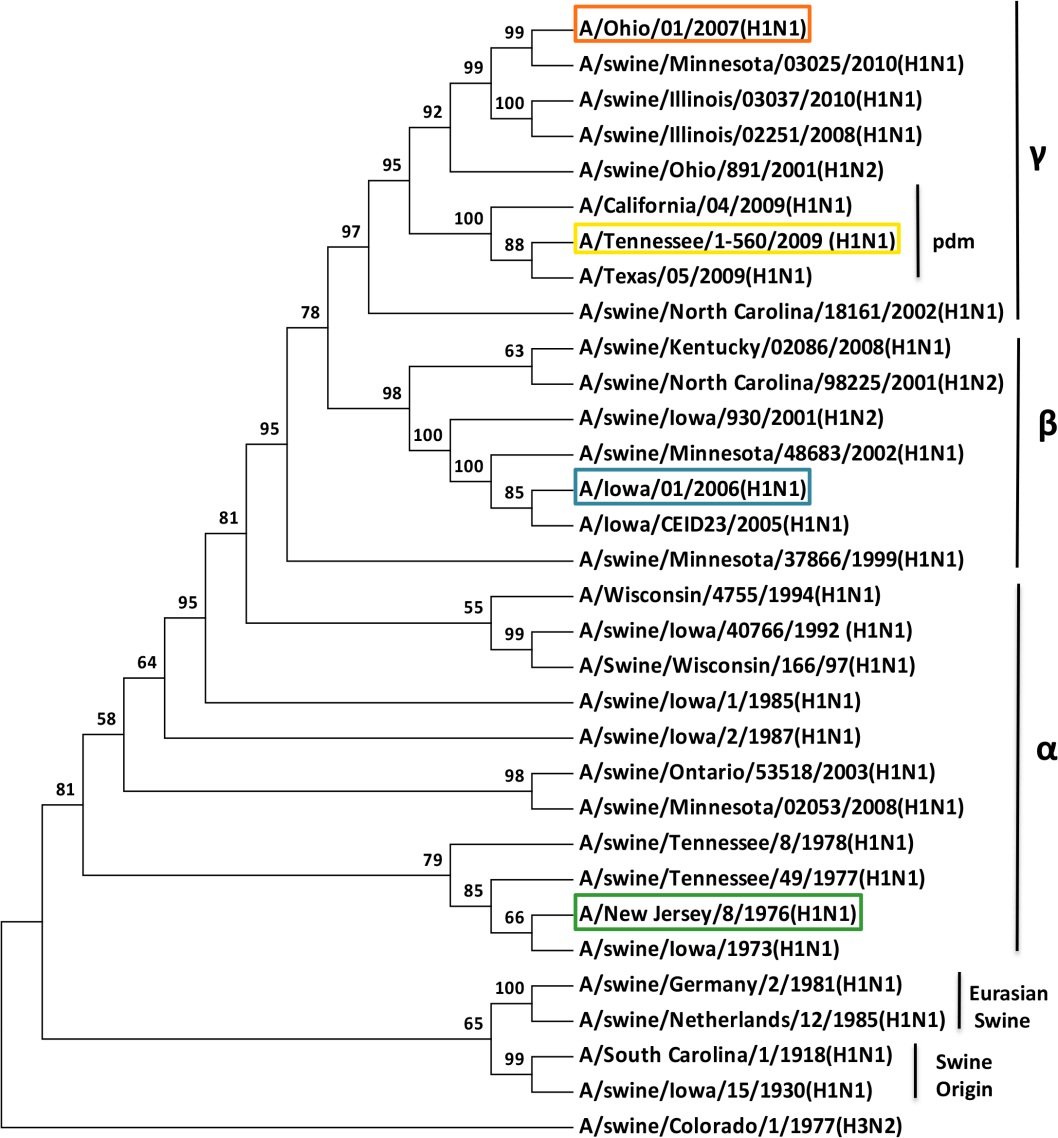
Phylogenetic comparison of swine H1 influenza hemagglutinins used to create the chimeric HAs. Parental viruses included in the DNA shuffling of chimeric HA genes are identified with colored boxes in each phylogenic clade. The phylogenetic tree was constructed using the Neighbor-Joining method by MEGA software version 6.0. The numbers on branches are bootstrap values from 1000 replicates.

Based on the phylogenetic analysis of influenza A H1 HA genes (Fig 1), four distinct parental influenza virus isolates were selected for generating chimeric HA constructs. Specifically, we selected A/Tennessee/1-560/2009 (TN09; 2009 human pandemic vaccine strain), A/Ohio/1/2007 (OH07; zoonotic isolate), A/Iowa/1/2006 (IA06; zoonotic isolate), and A/New Jersey/8/1976 (NJ76; zoonotic isolate included in the 1976 pandemic vaccine). Each of these represents one of the major phylogenetic clades of classical swine (α, β, and γ) and recent pandemic (pdm) strains, as defined previously [39,40]. Since analysis of influenza virus diversity based solely on genetic distance does not fully recapitulate the antigenic differences observed for influenza virus HA proteins, we performed a hemagglutination inhibition (HAI) assay to evaluate antibodies induced against each of the parental HA proteins expressed on a PR8 background. As shown in Table 2, reactivity of sera against homologous HA-expressing virus was at least four-fold higher than it was against heterologous HA-expressing viruses. The antigenic distance between the parental HA proteins was then calculated using the HAI titers, following criteria described by Cai et al [41]. As shown in Table 3, the closest antigenic distance for any of our selected parental HA proteins was 10-fold, which is greater than the four-fold antigenic difference that is used to define distinct isolates during vaccine selection. It is worth noting that the IA06 parental HA expressed on a PR8 background induced a strong antibody response against both the homologous HA and heterologous parental HAs (Table 2). Despite this high immunogenicity, the antigenic distance calculated for IA06 (Table 3) still indicated distinct antibody reactivity for this parental HA. Together, these data indicate that the viruses selected are both genetically and antigenically distinct.

**Table 2:** Antibody cross reactivity in sera from mice infected with recombinant viruses expressing parental or chimeric HA protein

Virus Isolate	Post-Infection Sera			
	OH07	IA06	NJ76	TN09
PR8-OH07	**1280**	320	*<	80
PR8-IA06	<	5120**	320	80
PR8-NJ76	<	320	**1280**	<
PR8-TN09	40	160	<	**320**
PR8_LAIV_-129	80	320	160	320

*<: HI titer less than 1:40.

** A four-fold difference in antibody reactivity represents an acceptable antigenic distance for vaccine selection [41].

**Table 3:** Antigenic distance between parental H1 HA proteins.

Virus Isolate	Antigenic distance			
	OH07	IA06	NJ76	TN09
PR8-OH07	NA*	136	256	18
PR8-IA06	136**	NA	10	18
PR8-NJ76	256	18	NA	160
PR8-TN09	18	18	160	NA

*NA: Not Applicable.

**A four-fold difference represents an acceptable antigenic distance for vaccine selection [41].

**Fig 2:**
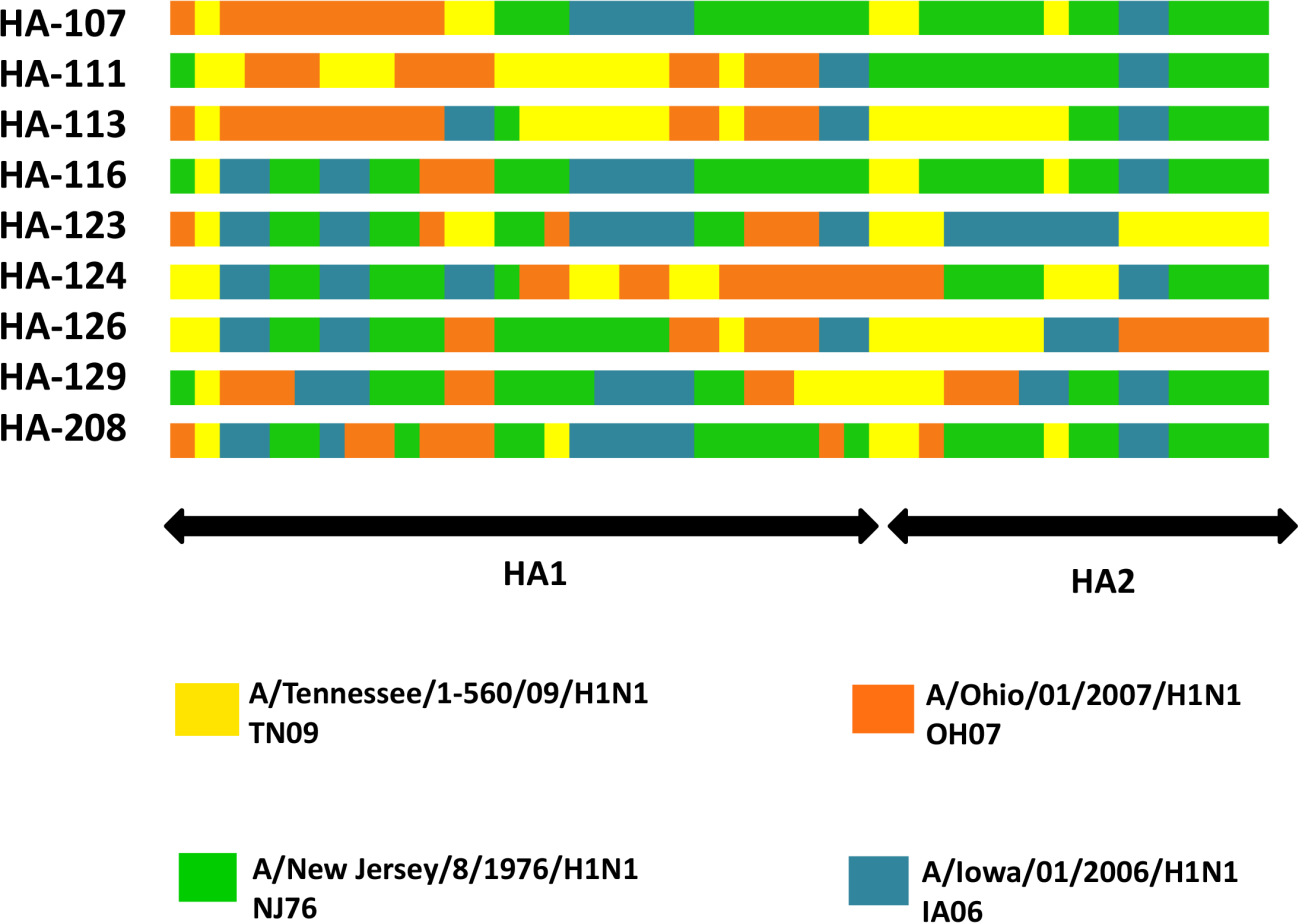
Schematic diagram of DNA shuffled chimeric HA gene sequences. Alignment of HA genes from chimeric constructs and parental viruses was performed using clustal W (MEGA 6) and the assignment of homology between each construct and the parental viruses was determined by a DNA shuffling alignment analysis tool (Salanto, version 2.0.2; https://bitbucket.org/benderc/salanto/wiki/Home). Different colors represent different HA gene elements from parental virus.

Subsequently, we constructed chimeric HA genes from these four parental viruses. HA genes were molecularly bred using the DNA shuffling method. A total of 33 chimeric HA genes were generated, and these shuffled HA constructs were cloned into the pHW2000 plasmid to establish an influenza HA antigen library. Using a previously created DNA shuffling alignment analysis tool [42], these HA constructs were evaluated for representative parental gene fragments within the chimeric sequence (Fig 2). Individual alignments were also created to compare the HA1 region of each parental HA with the chimeric HA constructs (S1–S4 Figs), which shows amino acids differences in the known antigenic sites and the receptor-binding site (RBS), as defined by others [39,43–45]. Nine constructs that contain the genetic elements from HA genes of all four parental viruses were selected for further analysis, including HA-107 (KR012992), HA-111 (KR012990), HA-113 (KR012994), HA-116 (KR012996), HA-123 (KR012995), HA-124 (KR012997), HA-126 (KR012998), HA-129 (KR012993), and HA-208 (KR012991).

### DNA vaccination with selected chimeric HA constructs in mice

**Fig 3:**
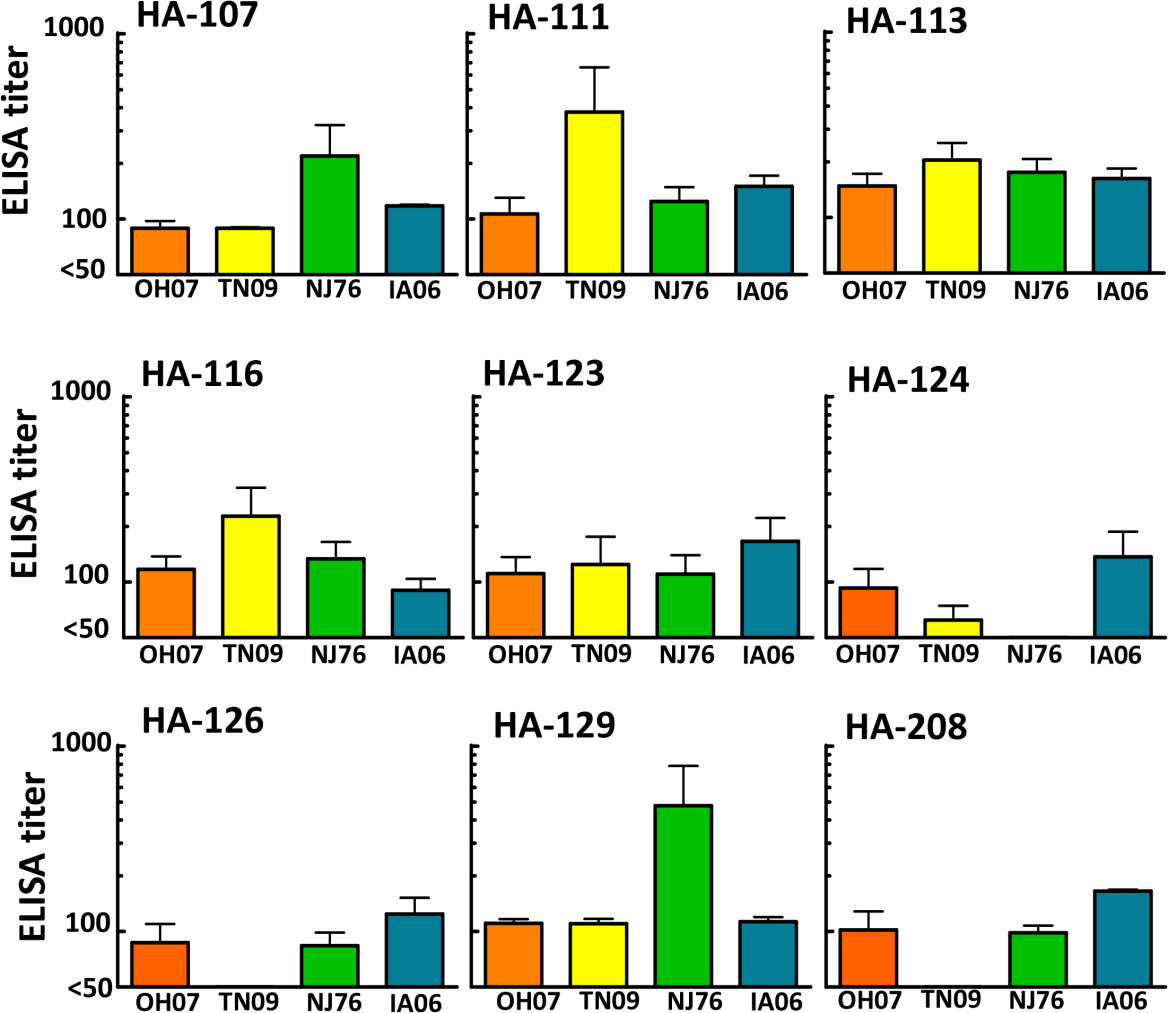
IgG antibody response in mice immunized with plasmid DNAs expressing chimeric HA. Mice (n = 4) were vaccinated with plasmid DNAs of chimeric HA, delivered by gene gun. Serum antibody (IgG) titers after third vaccination were evaluated by ELISA, with samples considered positive if their initial serum OD_405_ was at least three times greater than the OD_405_ of negative control sera. Samples with antibody titers below the detectable limit of the assay were assigned a titer of 50 for the purpose of generating graphs. Horizontal bars show mean values, and vertical error bars indicate standard deviation.

After screening the HA composition, selected chimeric HAs were screened in mice using DNA immunization. Serum samples collected at 14 days after a third inoculation with DNA were tested for antibody responses using an ELISA that incorporated parental HA-expressing viruses as antigen. The results show that IgG antibodies against all four parental viruses were detected in constructs HA-107, HA-111, HA-113, HA-116, HA-123, and HA-129 (Fig 3). Of note, the HA-124, HA-126, and HA-208 chimeras did not induce antibodies that consistently reacted with all four parental viruses. These data demonstrate that chimeric HA constructs created using DNA shuffling method have the ability to induce broad antibody responses, with some of these constructs inducing antibodies that react with all four parental HAs.

### Characterization of influenza viruses expressing chimeric HA genes

Historical approaches for influenza virus vaccine development utilize the natural reassortment properties of influenza viruses to express viral HA and NA genes on a PR8 master donor virus backbone [46,47]. Since the majority of commercial vaccine preparations still utilize this reassortment approach [48,49], we used reverse genetics to generate viruses for candidate vaccine production [29]. Efforts to create viruses expressing these chimeric HAs yielded only the HA-129 construct as an HA that could be expressed within a whole virus. This HA protein was expressed on both the PR8_LAIV_ (PR8_LAIV_-129) and the TX98 (TX98-129) backbones, which were further used for vaccination in mice and pigs, respectively.

**Fig 4:**
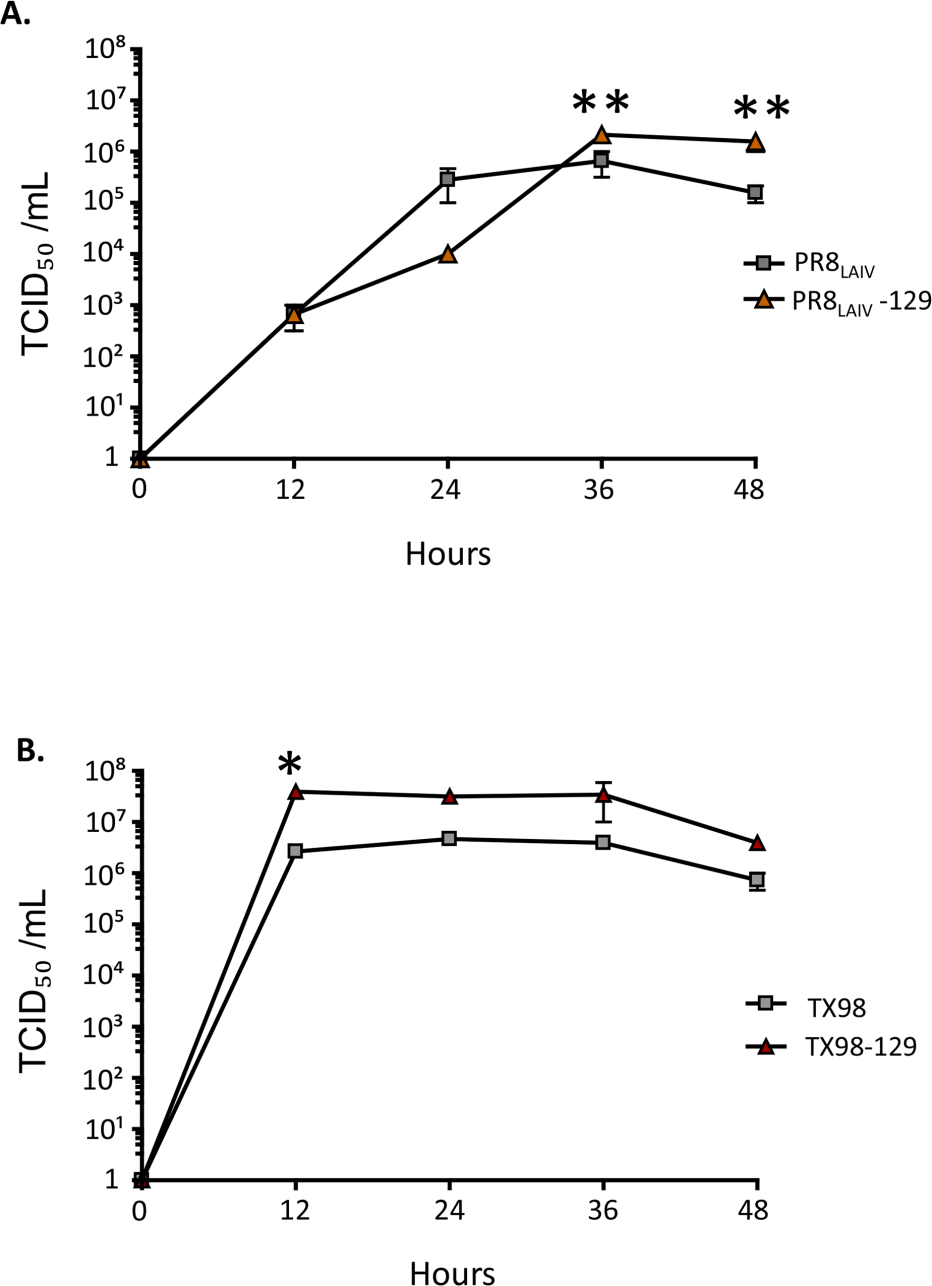
Comparison of growth kinetics of wild type virus with recombinant viruses expressing HA-129. (**A**) MDCK monolayers were inoculated with 0.01 MOI of either wild type virus PR8_LAIV_ or recombinant virus PR8_LAIV_-129. (**B**) MDCK monolayers were infected with wild type virus A/swine/Texas/4199-2/98 (H3N2) or recombinant virus TX98-129. At the 12-hour time points indicated, cell culture supernatants were collected, and virus titers were determined using TCID_50_ quantitation. Error bars represent SEM, with significance between paired viruses at time points denoted by asterisks (*p<0.05 and **p<0.01, using two-way repeated measures ANOVA with Bonferroni post-test).

To evaluate the *in vitro* properties of viruses expressing HA-129, we performed growth characterization of PR8_LAIV_-129 and TX98-129 in MDCK cells. Specifically, the growth kinetics of these recombinant viruses were compared with those of either PR8_LAIV_ or TX98 in MDCK cells. Supernatants were harvested from virus-infected cells every 12 hours for 48 hours post-inoculation, and TCID_50_ values were quantified at each time point by virus titration in separate MDCK cell monolayers. The results show that both PR8_LAIV_-129 and TX98-129 exhibit similar growth kinetics to that of PR8_LAIV_ (Fig 4A) and TX98 (Fig 4B), indicating that virus growth was not inhibited by the expression of HA-129 at the surface of these viruses. Similarly, in chicken eggs, both PR8_LAIV_-129 and TX98-129 grew to high titers, with TCID_50_ values of 10^8.375^mL^-1^ and 10^7.5^mL^-1^, respectively. Together, these data indicate that candidate whole virus vaccines expressing chimeric HAs can be propagated using either eggs or MDCK cells, without obvious deficiencies in growth characteristics.

### Antibody response induced by HA-129 in animal models

**Fig 5:**
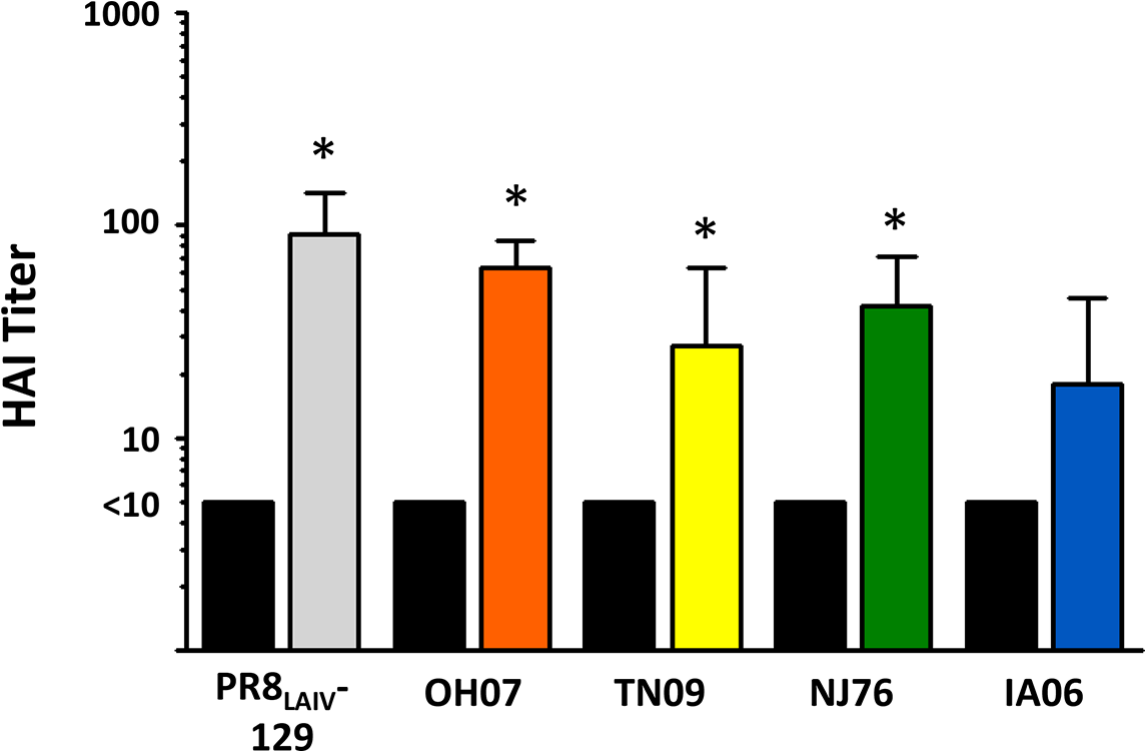
Serum antibody HAI titers from mice inoculated with recombinant virus PR8_LAIV_-129 vaccine. Balb/c mice (n = 7) were vaccinated intranasally with PR8_LAIV_-129. Serum antibody titers were analyzed using the HAI assay against the parental viruses and PR8_LAIV_-129 itself. HAI titers are defined as the reciprocal of the final serum dilution where inhibition of hemagglutination was observed. Serum samples with a titer below the detectable limit of the assay (initial serum dilution of 1:10) were assigned a value of 5 for graphical representation and statistical analyses. HAI titers from vaccinated (color bars) and unvaccinated (black bars) groups are presented for each HA tested (PR8_LAIV_-129, OH07, TN09, NJ76, and IA06). Reactivity of antibodies induced by PR8_LAIV_-129 from vaccinated mice was compared with that of unvaccinated mice (n = 7) using Mann Whitney nonparametric test (*p<0.05). Bars represent mean values for the indicated groups, with vertical error bars indicating standard deviation.

Using the PR8_LAIV_-129 as antigen, HAI assay results show that immune sera from mice inoculated with parental viruses broadly reacted with this chimeric HA-expressing virus (Table 2). To determine whether the PR8_LAIV_-129 can be used to induce broad immune responses, we thenvaccinated mice with this chimeric HA-expressing virus. Specifically, mice were immunized twice with the PR8_LAIV_-129, and sera were collected at 21 days post-secondary inoculation. Results from the HAI assay show that antibodies induced by the PR8_LAIV_-129 react with viruses expressing each of the four parental HAs, with maximal reactivity against the virus expressing the HA-129 itself (Fig 5). This result indicates that HA-129 is immunogenic when expressed within a whole virus, and that antibodies induced can react with all four parental HA proteins.

**Fig 6:**
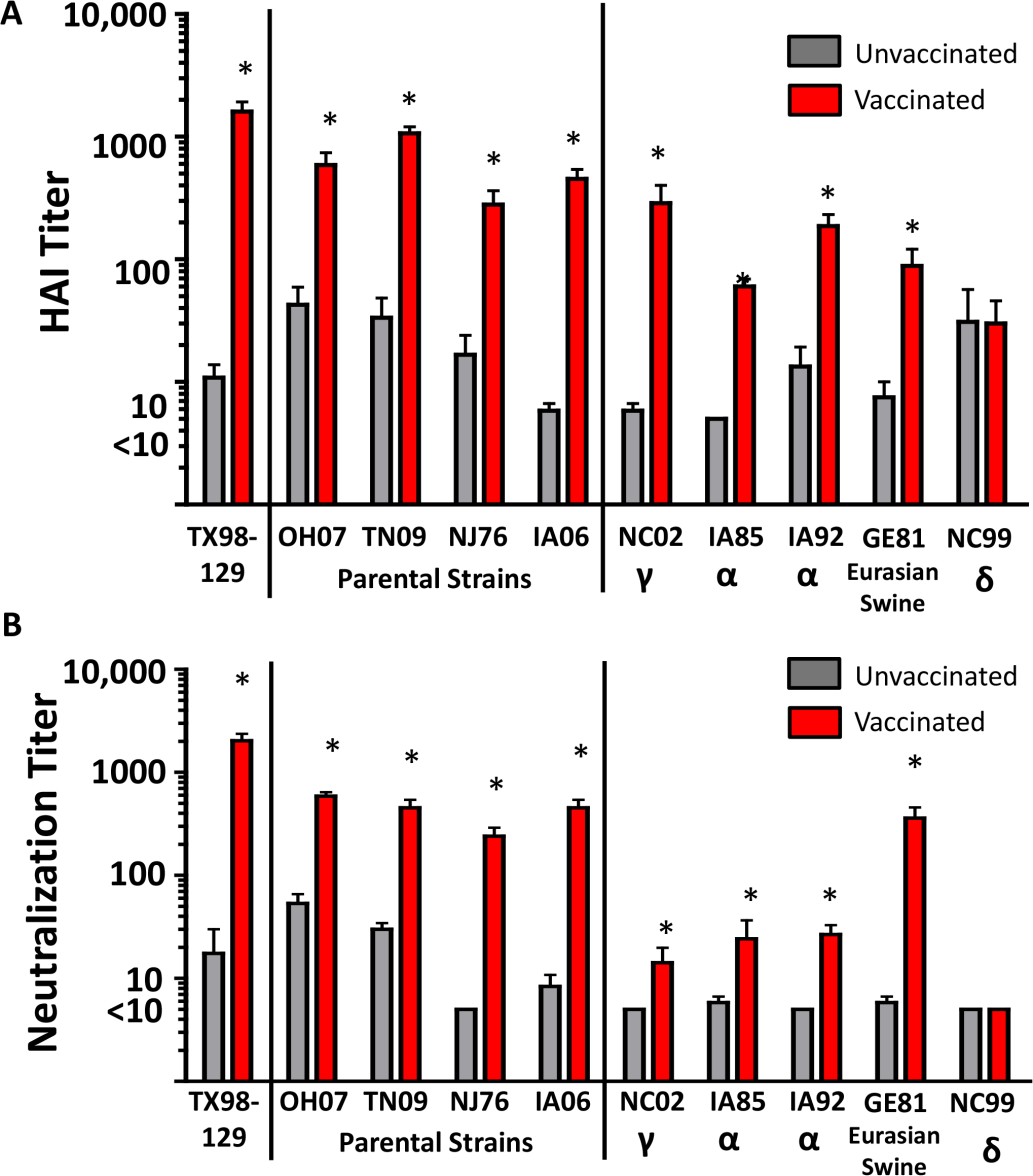
Antibody reactivity against viruses expressing parental or non-parental HAs using serum samples from pigs immunized with the TX98-129 IIV. Sera were collected at 14 days after secondary inoculation of pigs with the candidate TX98-129 IIV vaccine. (A) Serum antibody HAI titers are defined as the reciprocal of the final serum dilution where inhibition of hemagglutination was observed. (B) Serum antibody MN titers are defined as the reciprocal of the final serum dilution where OD_490_ was below 50% of positive control wells, using 100 TCID_50_ virus inoculum (confirmed by back-titration). In both panels, serum samples with a titer below the detectable limit of the assay (initial serum dilution of 1:10) were assigned a value of 5 for graphical representation and statistical analyses. Viruses expressing non-parental HA proteins are abbreviated (A/North Carolina/18161/2002: NC02; A/swine/Iowa/1/1985: IA85; A/swine/Iowa/40766/1992: IA92; A/swine/Germany/2/1981: GE81; A/New Caledonia/20/99: NC99) and shown with clade representation. Significance between vaccinated vs. unvaccinated for all viruses was determined using a Mann Whitney nonparametric test (*p<0.05).

To determine the immunogenicity of HA-129 in pigs, we immunized pigs with the formalin-inactivated TX98-129 virus. Serum samples were collected at 14 days post-secondary immunization for analysis using both HAI and MN assays (Fig 6). Similar to the results observed in mice, in both assays, immunized pigs developed increased antibody titers against the virus expressing HA-129, in comparison to the serum HAI and MN titers in unvaccinated control pigs. These vaccine-induced antibodies also showed reactivity against viruses expressing parental HAs from OH07, TN09, NJ76, and IA06. To further assess the breadth of immunity induced by HA-129, additional non-parental influenza virus variants representing the major phylogenetic clades within the H1N1 influenza A virus subtype were also tested. These results show that antibodies induced after vaccination with TX98-129 were significantly increased (p<0.05) against a non-parental γ clade variant (A/swine/North Carolina/ 18161/02, NC02), two additional α clade variants, A/swine/Iowa/1/85 (IA85) and A/swine/Iowa/40766/92 (IA92), and a virus from the Eurasian swine lineage (A/swine/Germany/2/81, GE81) [50], as detected using both HAI and MN assays. As a comparison, serum from TX98-129-vaccinated animals did not react with the A/New Caledonia/20/99 H1N1 virus (Fig 6), which was used here to represent the H1N1 δ clade [39]. This result is expected, since none of the parental viruses used for creation of HA-129 was from the δ clade.

## Discussion

In this study, we created a panel of chimeric HA constructs that have the ability to induce humoral immunity against four genetically divergent parental HAs. The parental viruses that we selected were isolated from zoonotic infections [25,51,52] and the 2009 pandemic cases [26]. Importantly, these viruses represent strains with the potential to cause future pandemics through genetic mutation. Our findings demonstrate that an HA-based, broadly-protective vaccine could be created using the DNA shuffling method, with the added benefit of incorporating these HA constructs into conventional virus vaccines that are immunogenic in both mice and pigs. Since the molecular breeding approach mimics and accelerates the natural evolutionary pathway, we hypothesize that the novel chimeric HA antigens created in this study could induce protective immunity against the current circulating H1 viruses, and that they may also have the ability to induce protective immunity against future emerging H1 strains. An influenza virus pandemic can emerge at any time, and current approaches for vaccine selection and production leave us 6–9 months away from a vaccine [12,53], we may not have a vaccine prepared to face the first wave of the next pandemic. Our data show that chimeric HA molecules can be constructed to improve the breadth of antibody responses within a single influenza A virus subtype (H1N1). This suggests that a vaccine developed using this approach might be able to limit the interspecies transmission of influenza viruses between pigs and humans, to either prevent a pandemic or at least lessen its impact.

In an effort to keep the vaccine development approach clinically relevant, we used reverse genetics to create viruses expressing chimeric HA constructs. We were able to successfully rescue recombinant viruses expressing the chimeric HA-129 on their surface (PR8_LAIV_-129 and TX98-129), and the growth kinetics analysis showed that expression of the HA-129 on either the PR8_LAIV_ or the TX98 genetic backbone did not affect virus propagation. The observation that these viruses could be propagated in eggs and MDCK cells provides a basis for future development of inactivated and live, attenuated influenza virus vaccine preparations using conventional, FDA-approved approaches for vaccine production [54]. The recombinant viruses rescued were used to vaccinate both mice and pigs, and they induced antibody responses against viruses expressing both parental and non-parental HAs in pigs. These data suggest that broad, protective immunity could be induced within the swine population using this chimeric HA construct. These results encourage our approach toward vaccinating pigs in the pre-pandemic phase, a practice that could be helpful for limiting interspecies transmission.

While not identical, the antibody response induced after DNA vaccination allowed us to screen our HA constructs individually, and could also predict the breadth of humoral immunity induced by the PR8_LAIV_-129 in mice and TX98-129 in pigs. This approach was in contrast to our prior attempt to induce broad immunity within an HA subtype by vaccinating with multiple HAs simultaneously. Specifically, the results reported here demonstrate advantages of using a single HA construct, instead of multiple parental HAs delivered simultaneously, especially when attempting to deliver these HAs in the context of a PR8_LAIV_ backbone [14]. Furthermore, the antibody response to PR8_LAIV_-129 in mice correlated with the antibody response against TX98-129 in pigs, in which significant levels of antibodies against TN09, NJ76, OH07, and IA06 HAs were generated. In fact, based on previous reports [55,56], a HAI titer of 1:40 is considered an accepted antibody level that correlates with protective immunity in both pigs and humans, and our TX98-129-vaccinated animals developed antibodies against all four parental HAs that either met or exceeded this level. However, some of the serum samples from unvaccinated pigs showed unexpectedly high reactivity in the HAI assay, so we further analyzed the serum using MN assay to confirm that the antibodies detected by HAI were indeed neutralizing. Similar to our results from the HAI assay, we observed significant differences in neutralizing antibody titers when comparing vaccinated and unvaccinated serum samples by MN. Together, our results demonstrate that DNA vaccination can be used as a tool for screening the breadth of immunity induced by chimeric HA gene constructs, and that immunity induced by whole virus vaccine preparations expressing chimeric HAs in mice could predict the performance of similar vaccines in pigs.

It is worth noting that some other HA chimeras, including HA-107, HA-111, HA-113, HA-116 and HA-123, induced broad antibody responses against all four parental HAs, even though we were unable to generate viable recombinant viruses when placing these HAs into the reverse genetics system. We attempted to identify unique epitopes expressed by these chimeric HA antigens using sequence alignments to analyze the amino acids difference between wild type and the shuffled HAs (S1–S4 Figs). However, this did not provide a direct, apparent clue on specific amino acids and/or epitopes that are associated with the increased breadth of immunity observed. Future, in-depth analyses will be required to narrow down the antigenic sites expressed by these chimeric HAs, and to identify the key amino acids/epitopes where the chimeric HA genes are specifically mutated. These future studies could ultimately lead to the design of HA constructs expressing critical epitopes in a manner that would allow us to create additional vaccines by reverse genetics, similar to the HA-129.

In addition to the effort to express novel HA epitopes within whole virus vectors, it is worth noting that in the time since this study was initiated, the FDA has approved the use of a recombinant protein vaccine that incorporates the influenza HA0 (rHA0) propagated in insect cells. This vaccine, known as FluBlok (Protein Sciences Corp., Meriden, CT), is now approved for use in adults aged 18–49 [57]. This approval of a recombinant protein-derived HA0 increases the potential application of our novel chimeric HA constructs as rHA0-like vaccines. This approach would increase the number of laboratory-derived chimeric HAs that could be tested in mice, pigs, ferrets, and ultimately humans, using a FDA-approved vaccine production technology.

The current study established a proof of concept and platform for creating novel HA genes of influenza viruses using the molecular breeding approach, and allowed for evaluating one of these constructs in animal models using relevant, whole virus vehicles. Importantly, the HA-129 expressed within virus particles was immunogenic in both mice (PR8_LAIV_-129) and pigs (TX98-129). Future work can be expanded to apply this molecular breeding approach either within or between the other influenza A virus subtypes that have pandemic potential, including H5, H7, and H9 viruses, which could have important implications in future development of broadly protective seasonal and pandemic influenza vaccines.

## Supporting Information

S1 Fig Comparison of antigenic sites of shuffled chimeric HA sequences with OH07 HA. Amino acids alignment comparing the individual parental HA of OH07 with chimeric HAs created in this study. Antigenic sites Ca1, Ca2, Cb, Sa, Sb, and the receptor binding site (RBS) were identified previously [39,43–45], and are indicated in the figure.
(PPTX)

S2 Fig Comparison of antigenic sites of shuffled chimeric HA sequences with TN09 HA. Amino acids alignment comparing the individual parental HA of TN09 with chimeric HAs created in this study. Antigenic sites Ca1, Ca2, Cb, Sa, Sb, and the receptor binding site (RBS) were identified previously [39,43–45], and are indicated in the figure.
(PPTX)

S3 Fig Comparison of antigenic sites of shuffled chimeric HA sequences with NJ76 HA. Amino acids alignment comparing the individual parental HA of NJ76 with chimeric HAs created in this study. Antigenic sites Ca1, Ca2, Cb, Sa, Sb, and the receptor binding site (RBS) were identified previously [39,43–45], and are indicated in the figure.
(PPTX)

S4 Fig Comparison of antigenic sites of shuffled chimeric HA sequences with IA06 HA. Amino acids alignment comparing the individual parental HA of IA06 with chimeric HAs created in this study. Antigenic sites Ca1, Ca2, Cb, Sa, Sb, and the receptor binding site (RBS) were identified previously [39,43–45], and are indicated in the figure.
(PPTX)
