# Supplementary figures and images for: Construction and Immunogenicity Evaluation of Recombinant Influenza A Viruses Containing Chimeric Hemagglutinin Genes Derived from Genetically Divergent Influenza A H1N1 Subtype Viruses
Source: PLoS One. 2015 Jun 10;10(6):e0127649. doi: 10.1371/journal.pone.0127649 (PMC4465703; doi:10.1371/journal.pone.0127649)

## Slide 1
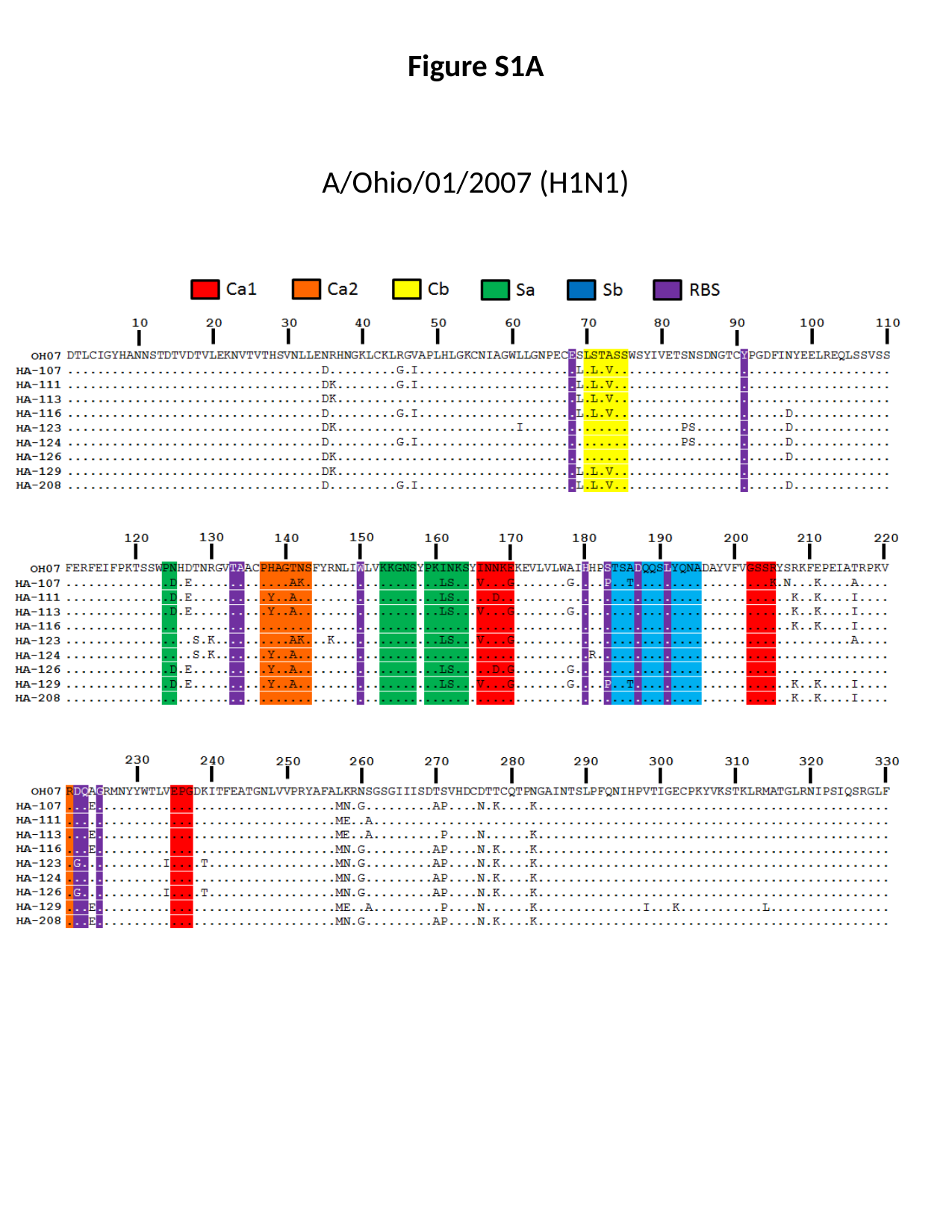

Figure S1A
A/Ohio/01/2007 (H1N1)

Supplement: S1 Fig — Amino acids alignment comparing the individual parental HA of OH07 with chimeric HAs created in this study. Antigenic sites Ca1, Ca2, Cb, Sa, Sb, and the receptor binding site (RBS) were identified previously [39,43–45], and are indicated in the figure. (PPTX) [file pone.0127649.s001.pptx]

## Slide 1
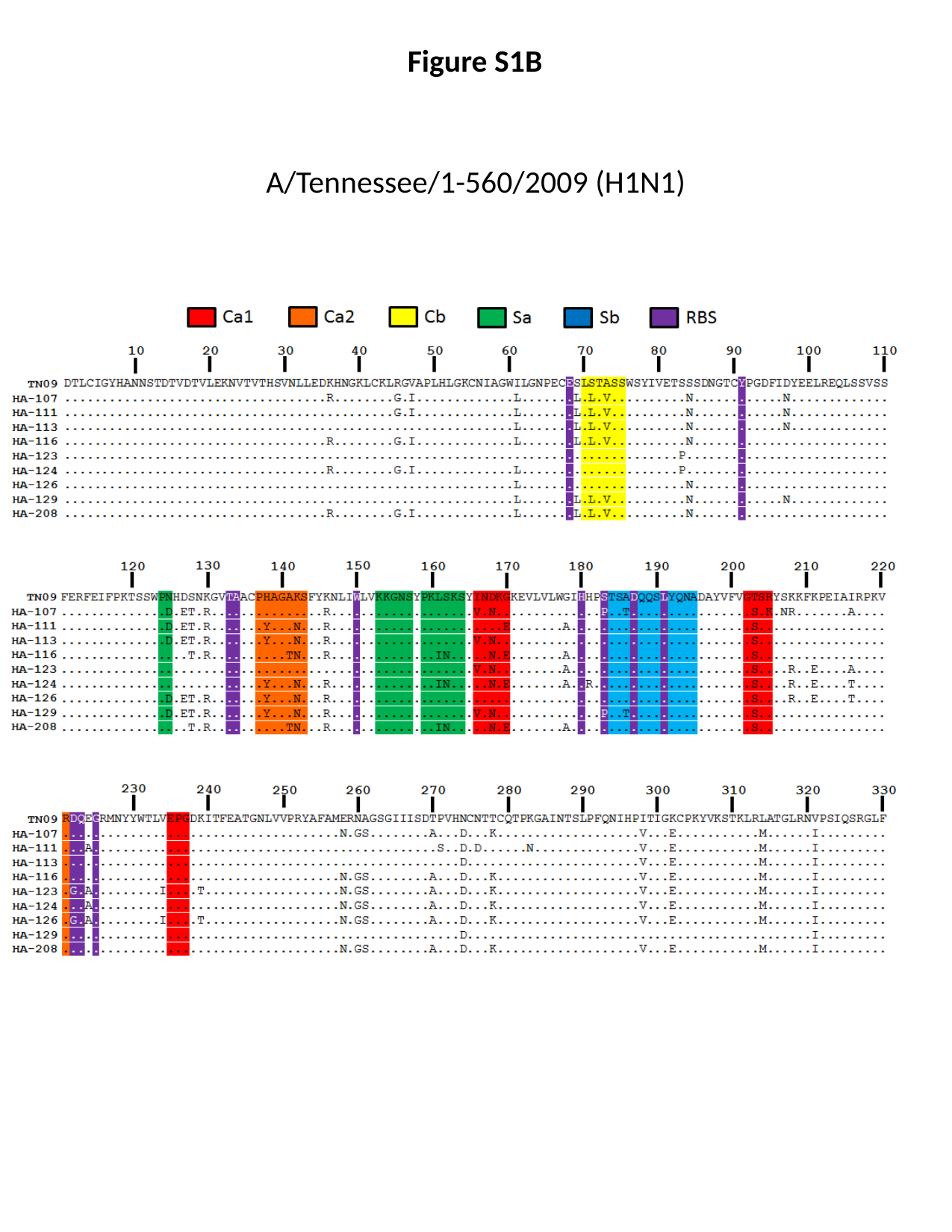

Figure S1B
A/Tennessee/1-560/2009 (H1N1)

Supplement: S2 Fig — Amino acids alignment comparing the individual parental HA of TN09 with chimeric HAs created in this study. Antigenic sites Ca1, Ca2, Cb, Sa, Sb, and the receptor binding site (RBS) were identified previously [39,43–45], and are indicated in the figure. (PPTX) [file pone.0127649.s002.pptx]

## Slide 1
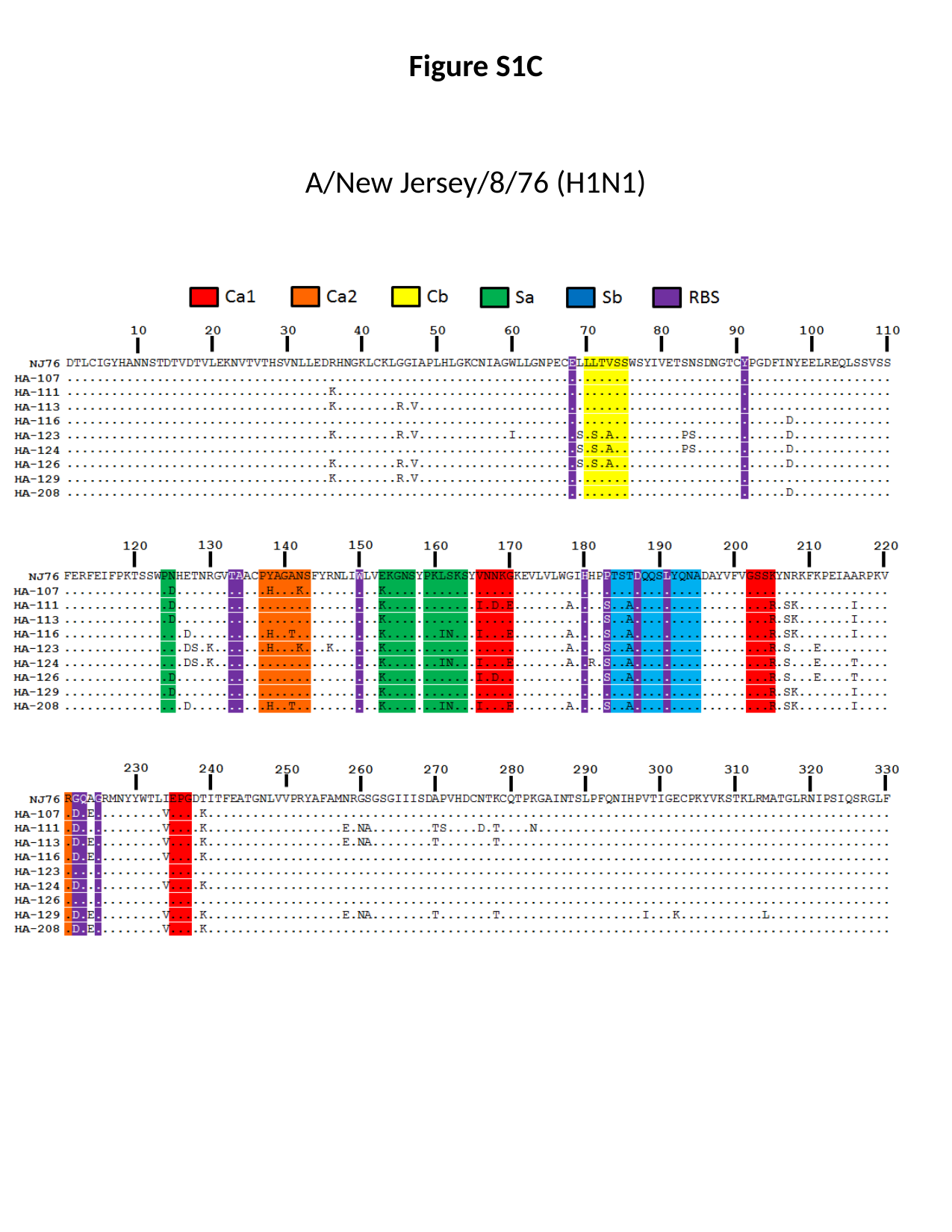

Figure S1C
A/New Jersey/8/76 (H1N1)

Supplement: S3 Fig — Amino acids alignment comparing the individual parental HA of NJ76 with chimeric HAs created in this study. Antigenic sites Ca1, Ca2, Cb, Sa, Sb, and the receptor binding site (RBS) were identified previously [39,43–45], and are indicated in the figure. (PPTX) [file pone.0127649.s003.pptx]

## Slide 1
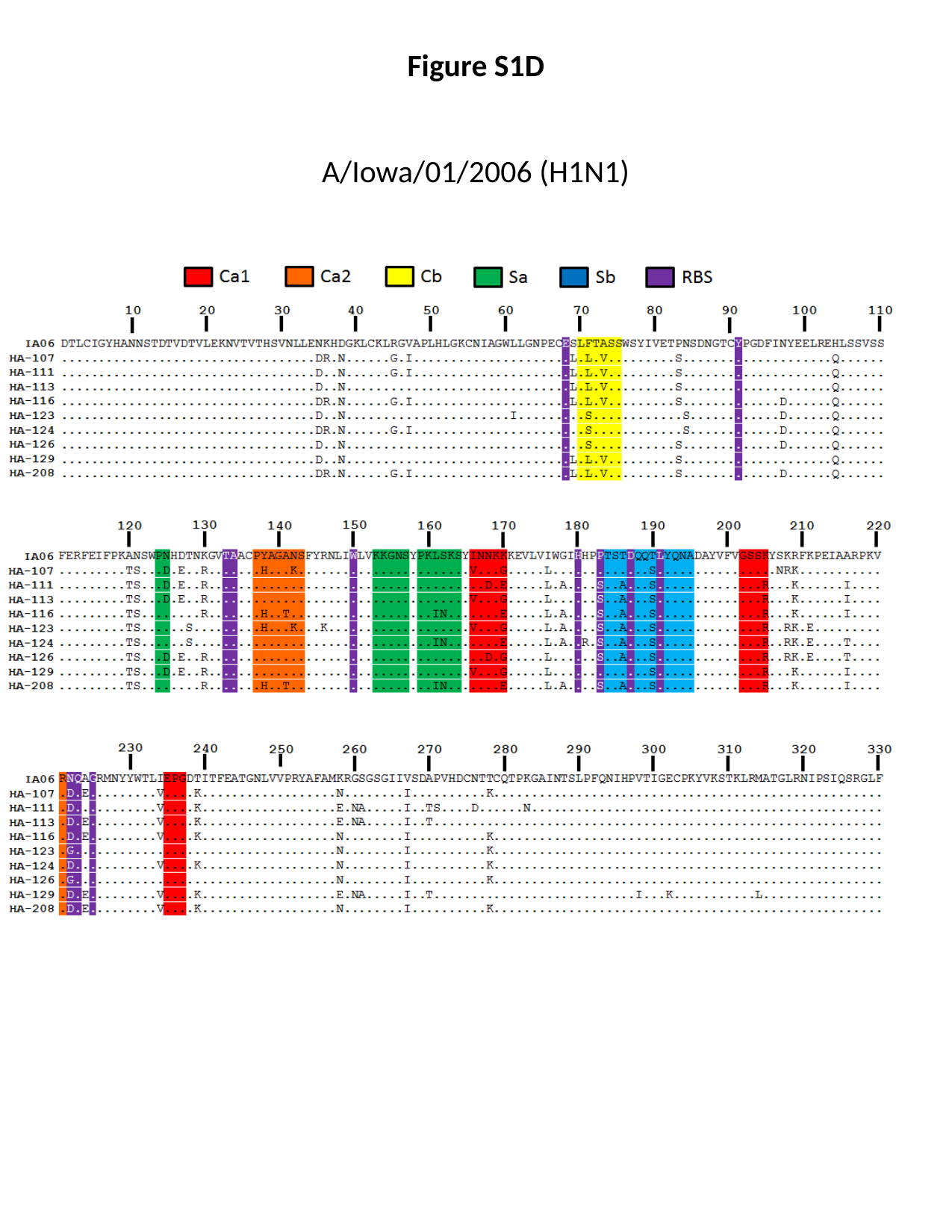

Figure S1D
A/Iowa/01/2006 (H1N1)

Supplement: S4 Fig — Amino acids alignment comparing the individual parental HA of IA06 with chimeric HAs created in this study. Antigenic sites Ca1, Ca2, Cb, Sa, Sb, and the receptor binding site (RBS) were identified previously [39,43–45], and are indicated in the figure. (PPTX) [file pone.0127649.s004.pptx]
